# Understanding sexual and reproductive health needs of adolescents: evidence from a formative evaluation in Wakiso district, Uganda

**DOI:** 10.1186/s12978-015-0026-7

**Published:** 2015-04-22

**Authors:** Lynn M Atuyambe, Simon P S Kibira, Justine Bukenya, Christine Muhumuza, Rebecca R Apolot, Edgar Mulogo

**Affiliations:** Department of Community Health and Behavioural Sciences, Makerere University School of Public Health, P.O.Box 7072, Kampala, Uganda; Department of Epidemiology and Biostatistics, Makerere University School of Public Health, Kampala, Uganda; Department of Community Health, Mbarara University of Science and Technology, Kampala, Uganda

**Keywords:** Adolescent, Sexual, Reproductive health, Needs, Uganda

## Abstract

**Introduction:**

Adolescents are frequently reluctant to seek sexual and reproductive health services (SRH). In Uganda, adolescent health and development is constrained by translation of the relevant policies to practice. Recent studies done in central Uganda have shown that there is need for a critical assessment of adolescent friendly services (AFS) to gain insights on current practice and inform future interventions. This study aimed to assess the sexual reproductive health needs of the adolescents and explored their attitudes towards current services available.

**Methods:**

A qualitative study was conducted in Wakiso district, central Uganda in September 2013.Twenty focus group discussions (FGDs) stratified by gender (10 out-of-school, and 10 in-school), were purposefully sampled. We used trained research assistants (moderator and note taker) who used a pretested FGD guide translated into the local language to collect data. All discussions were audio taped, and were transcribed verbatim before analysis. Thematic areas on; adolescent health problems, adolescent SRH needs, health seeking behaviour and attitudes towards services, and preferred services were explored. Data was analysed using *atlas ti* version 7 software.

**Results:**

Our results clearly show that adolescents have real SRH issues that need to be addressed. In and out-of-school adolescents had sexuality problems such as unwanted pregnancies, sexually transmitted infections (STIs), defilement, rape, substance abuse. Unique to the females was the issue of sexual advances by older men and adolescents. We further highlight RH needs which would be solved by establishing adolescent friendly clinics with standard recommended characteristics (sexuality information, friendly health providers, a range of good clinical services such as post abortion care etc.). With regard to health seeking behaviour, most adolescents do not take any action at first until disease severity increase.

**Conclusions:**

Adolescents in Uganda have multiple sexual and reproductive health needs that require special focus through adolescent friendly services. This calls for resource support in terms of health provider training, information education and communication materials as well as involvement of key stakeholders that include parents, teachers and legislators.

**Electronic supplementary material:**

The online version of this article (doi:10.1186/s12978-015-0026-7) contains supplementary material, which is available to authorized users.

## Introduction

Currently, there are more than one billion adolescents 10-19 year, 70% of whom live in low income countries [1,2]. It is, therefore, critical for countries to engage with this significant portion of the population and be able to address their health needs. Health services need to move beyond adolescent pregnancy and HIV to address the full range of adolescents’ health and development needs [3].Adolescents develop more self-consciousness indicated in their self-assessment of how others see them [4].In many cases adolescents consider themselves grown up and mature enough to have sex [5-7] yet they have inadequate knowledge about the consequences of unprotected sex. These consequences include unwanted pregnancy, complications of unsafe abortion, and sexually transmitted infections [8-10]. In many cases, they do not reveal their reproductive health problems and tend not to use the healthcare services they actually need [11]. This may be due to inadequate information, limited access to financial resources or negative attitudes of health workers [12,13]. Reproductive health of adolescents is dependent on several complex and often independent factors including social-cultural influences (such as family, peers and communities), and access to health services, education and employment opportunities [14]. In Uganda abortion is legally restricted and post abortion care (PAC) services are provided by doctors, clinical officers and midwives in all facilities [15]. The provision of PAC services is always limited by inadequate trained staff and lack of other resources.

Although both male and female adolescents have many reproductive health challenges, the female adolescents have additional burdens that are gender and sex specific. For example, in the year 2011, 24 percent of adolescents 13-19 years were already mothers or pregnant with their first child in Uganda [16]. Even though adolescent pregnancy has been steadily declining (41% in 1995, 31% in 2000, 25% 2006 and 24% in 2011), it is still visibly high [16,17]. Pregnancy among adolescents is important because it is associated with higher morbidity and mortality not only for the mother but also the child. It also has the psycho-social consequences that affect their wellbeing [18-20].In countries such as Ethiopia and Uganda, early marriage often fuels high incidence of complications from pregnancy and delivery [21]. Moreover, a growing number of young people are becoming sexually active before marriage and as a consequence the rate of unplanned pregnancies among this age group, particularly among those with unmet need for contraceptives increases [21,22].The damaging consequences of child bearing at a young age pose health threats to both the adolescent mother and infant. Adolescent sexual activity, within or outside of marriage, can lead to negative reproductive health outcomes [23].

Adolescents are quite explicit about what they want from health-care providers. They value their privacy and identity, and want to make decisions for themselves based on correct information. WHO stipulates a number of elements that stimulate adolescents to seek healthcare.These elements include: confidentiality, provision of required information and services, accepting adolescents as they are, considering and respecting adolescents’ opinions, allowing adolescents to make their own decisions, ensuring that adolescents feel welcome and comfortable, being non-judgmental, and provision of services at a time that adolescents are able to come. In Uganda, girls become sexually active earlier than boys. In 2011, the median age of first sexual relationship for women aged 25 to 49 years was 16.8 years compared with 18.6 years for men [16]. Adolescents are frequently reluctant to seek health services for sexual and reproductive health. Included among the many barriers are judgmental health workers, lack of supplies, equipment, materials and private workspace, and a lack of training for and in understanding of adolescent reproductive needs [24-26]. The policies in Uganda are favourable for adolescent health and development. The main limiting factor is the translation of these policies into practice. Uganda has Adolescent Health Policy Guidelines and Service Standards [27], the *National Minimum Healthcare Package* which includes sexual reproductive health (SRH) and rights for adolescent [28], and the Health Sector Strategic and Investment Plan (HSSIP), MoH 2010.Recent studies done in the same district contend that there is a need to take a critical assessment of AFS in order to provide appropriate interventions [29] .A survey on female adolescent sexuality in Kenyan schools revealed a high risk of early pregnancy and induced abortion and many had inadequate sexual and reproductive health knowledge [30].

While half of Uganda’s population consists of adolescents, adolescent sexual and reproductive health services are limited and do not address the needs of adolescents (HSSIP, 2010). In-spite of the conducive policy environment, the HSSIP, 2010-15 points out that the proportion of health facilities that are adolescent-friendly are only 10% but need to be increased to 75% by 2015. Other targets include reducing adolescent pregnancy rate from 24% to 15% by 2015. The government of Uganda recognises a need to strengthen adolescent sexual and reproductive health services. Some of the ways identified to achieve this would be to: avail updated information education and communication materials on adolescent health and development, integrate and implement adolescent sexual and reproductive health in school health programmes, and increase the number of facilities providing adolescent friendly sexual and reproductive health services. Service providers should be trained and accessible, respect adolescent sexual and reproductive health rights and be non-judgmental. Besides, the facilities themselves should be conveniently located with adequate space to promote adolescent participation in service delivery with a comfortable environment that offers both visual and auditory privacy with gender sensitive sanitation facilities. This study therefore aimed to assess the sexual reproductive health needs of the adolescents and explored their attitudes towards current services available.

## Methods

### Design and study area

This was a qualitative study using focus group discussions (FGDs) among adolescents aged 10 to 19 years in Wakiso District, central Uganda. The district population in 2013 was1,429,500, with 52% women and an average household size of 4.1persons [31]. Young people (10-24 years) comprise over one third of that population. Most of the population is rural while the small urban and peri urban population is heavily influenced by city lifestyle with proximity to Kampala city. Wakiso has two counties and one municipality, 17 sub-counties and 131 parishes.

### Study methods, selection of study participants and data collection

We conducted 20 FGDs in the two counties of Kyaddondo and Busiro. We then randomly selected three sub counties from each of the two county ensuring inclusions of both peri urban and rural localities. In each sub county, we conducted four FGDs with adolescent girls as well as boys (in and out of school) irrespective of whether they were seeking care or not (Table 1).

**Table 1:** **Adolescent FGD participant’s social demographic characteristics**

**County**	**Sub-county**	**FGD categories**	**Sex**	**Average age**	**Average level of education completed**	**Number of participants**	**No of FGDs**
Busiro	Namayumba	In-School	Male	16.5	Senior three	8	1
		Out-School	Male	17	Senior one	7	1
		In-School	Female	15.5	Senior three	9	1
		Out-School	Female	17	Primary six	7	1
	Sub-total						**4**
	Sissa	In-School	Male	15.5	Senior two	8	1
		Out-School	Male	18	Primary six	7	1
		In-School	Female	16.5	Senior three	8	1
		Out-School	Female	17	Primary seven	6	1
	Sub-total						**4**
Kyadondo	Nangabo	In-School	Male	16	Senior four	7	1
		Out-School	Male	18	Senior one	7	1
		In-School	Female	15.5	Senior three	9	1
		Out-School	Female	18	Primary five	7	1
	Sub-total						**4**
	Ndejje	In-School	Male	18	Senior four	9	1
		Out-School	Male	19	Primary five	8	1
		In-School	Female	16.5	Senior three	9	1
		Out-School	Female	18	Primary seven	7	1
	Sub-total						**4**
	Wakiso T/C	In-School	Male	18	Senior four	9	1
		Out-School	Male	17.5	Senior one	7	1
		In-School	Female	17	Senior three	9	1
		Out-School	Female	18.5	Senior two	8	1
	**Sub-total**						**4**
**Grand total**							**20**

This selection was to ensure intra group homogeneity since the two groups of adolescents have been shown to exhibit different behaviours regarding health [32]. The FGDs were conducted within communities where the different categories of adolescents were identified. Through FGDs, we explored the SRH issues that affect the adolescents and their health seeking behaviours and attitudes towards available SRH services. We recruited two experienced research assistants who also had a good working knowledge of English, and the local language (Luganda) and trained them in data collection for this study. Investigators also actively participated in the data collection process. The FGD guides were translated from English to Luganda, then back translated and compared to ensure consistency. The FGD guides were pre-tested in Kampala district, Central Uganda. FGDs lasted on average one and half hours and all were audio recorded with consent.

### Data management, analysis and ethical consideration

The FGDs were transcribed verbatim and those in the local languages translated carefully not to alter meaning. We read the typed transcripts several times, developed codes and code definitions based on the objectives of the study. Codes were grouped into categories and then themes identified as stipulated by Graneheim and Lundman, 2004 [33]. We coded and analysed the data using *atlas ti* qualitative data analysis software version 7 (atlasti.com). We analysed the coded transcripts by running query reports and primary documents tables of codes by objective (theme), carefully teasing out key messages and code counts to explore the magnitude of issues from the various FGDs by category. Quotes illustrating meaning or key message from the analysis were selected based on code count or how illustrative to the theme or sub-theme the quotes were.

Ethical clearance was obtained from Makerere University School of Public Health Research and Ethics Committee and approval from the Uganda National Council of Science and Technology (UNCST), study number SS 2894. Permission to carry out the research was further sought from the Wakiso district health office and the management of the respective health facilities. The objectives, benefits and risks of the study were explained to the study participants and written informed consent obtained from adolescents above 18 years and those below 18 years but who were pregnant or had given birth (these are categorised as emancipated minors by the ethical review committees). Parental/guardian assent was sought for adolescents aged10 to17 years and their own consent before the FGDs were held. All data obtained during the study were treated confidential and anonymous identifiers used. We restricted data access to only the investigators and the two research assistants.

## Results

The results are presented in fourthematic areas originating from the data analysis namely; i) main adolescent health problems, ii) adolescent SRH needs, iii) health seeking behaviourr and attitudes towards services, and iv) preferred services and modalities for their provision.

### Main health problems affecting adolescents

Adolescents (female and male) in and out of school described multiple issues concerning their sexual and reproductive health needs. The most important health problems expressed were HIV/STIs (all FGDs), unwanted pregnancies, sexual advances for the females from adult males and fellow male adolescents (expressed by female adolescents mostly), defilement and rape and the use of alcohol and other substances.

*Most of the youths don’t have what to do - they resort to taking alcohol, opium, cigarettes and marijuana. Even girls think that it is the today’s style to put on thin trousers called ‘skin tight’. And in so doing they meet these boys who have taken opium along the roadside and they force them into sex. In every town there are places like clubs and bars where youths take alcohol.* (Males out of school)

### Main adolescent reproductive health needs

Various adolescent Reproductive Health (RH) needs were reported in all FGDs ranging from condoms, need for youth counsellors, teenage medical centres and post abortion care services. Whereas nine of the ten males FGDs highlighted the need for male condoms to be put in accessible places at no cost, the need for a youth counsellor was expressed by eighteen of the twenty females and males FGDs.

The participants in both female and male FGDs reported post abortion care services as a great need that was not catered for. In some FGDs especially those with in school adolescents, they reported knowledge of their colleagues in schools and out of school who had used traditional dangerous methods and other unsafe means to get rid of unwanted pregnancies even with knowledge of consequences associated with these means including death. Some also reported that some health workers give them medicines to take home in case they want to abort or even help with illegal abortions.

*I was told that there are some girls who get some medicines from health workers. It is in form of tablets. When they swallow it, it gives them labour like pain, then they deliver and the pregnancy is terminated.* (Males out of school)

*Another problem I see as a young person is among the girls, they have aborted a lot. They don’t get good counselling or advice on how they can protect and take care of the pregnancies. So they end up aborting.* (Males in school)

Infrastructure providing exclusive services for teenagers was reported as lacking in the majority (seventeen of the twenty) of FGDs. Some male adolescents said that it was always hard for them to collect condoms and other medicines from the general health facilities where all the adults including their parents and relatives receive care from. However, they expressed willingness to freely do so if they had a separate medical centre.

*…[we need a teenage centre] for even medical check-ups, so that you know that if you are not feeling safe there is a centre where you can go for check-up and know your HIV status, or Candida [STIs] or if you are pregnant. That is better than these services which cost money where you may even be afraid and you take about 3 years without testing.* (Females in school)

There was an expressed wish about the staff working in such proposed centres to be of some age range or at least trained to handle youth problems according to the adolescents’ perception.

*Youth centre as you hear that word youth centre, which means even the workers must be youths. It will be easy to consult them about what happened to me and they tell me. It means there will be family planning, their use and their effects.* (Males in school)

Regarding contraceptive access, the issue of condom availability and cost was highlighted as a main problem especially by the males in nine out of ten male FGDs and the females in school. Only two out of the five out-of-school female expressed the condom problem.

*The problem we have pertaining condoms e.g. life guard, some are expensive especially in the shops and the cheap ones are not available. So we young people end up having sex without condoms. (*Males in school)

Our results further point out some gaps related to sex education reported by a few male adolescents but that could be very important to ignore. For example within health centres, it was pointed out that in some cases health workers distribute condoms without accompanying health education on their use.

*…the need for condoms, you can go to the health facility and they give them to you but there are some people who do not know how to use them. He will just put it on anyhow. It requires that there is someone who gives them out but he/she first educated you on how to use them and he tells you that “I have given you that thing, use it like this. But there are those who just have them for showing off; they tell their friends that they have them.* (Males out of school)

Sexual activity was reportedly high among the adolescents and thus a need for contraceptive education and provision. Most female FGDs expressed a need to provide family planning education including the natural methods as highlighted below.

*And another thing, is the need to educate them [adolescents] how to count those menstrual days in the cycle though they keep on changing say for one month you have periods on 5*^*th*^*and the second month you experience periods on 3*^*rd*^*. Because some of the girls use those safe days but they don’t know how to count them for example like this one has said she doesn’t know the day she goes into her MPs. But if you health educate here, say you tell her that since you started this month on 3*^*rd*^*the next month you will experience your periods on this date. So they will know.* (Females in school)

*We also need things that can prevent us from HIV/AIDS and also we need pregnancy control things like condoms, pills, coils, injectaplan, so that it can save some youths. Even counselling is needed.*(Females out of school)

Regarding available services adolescents reported that condoms, male circumcision services and post abortion care services were the least available services in health facilities at the community level while HIV Counselling and Testing was more available.

*[we need post abortion services], my cousin for example got an unwanted pregnancy and she went to one of the health workers in a clinic to abort and the health worker performed some procedures, told her to go back home and after the two to three hours to go where she wants to drop that foetus and it will come out. So she squat on a bucket and it came out but she felt a lot of pain for about 2 weeks and it was associated with a lot of bleeding. She is fine now but sometimes still experiences pain inside her stomach. [Such people] when they want to abort, should be directed the right ways of aborting so that they don’t feel that pain.* (Females in school)

### Health seeking behaviour of adolescents and attitudes towards available services

We assessed the health seeking behaviours of adolescents for RH needs. In all FGDs, we asked adolescents what they and other adolescents do when they have RH needs. Our findings show that some adolescents when faced with RH problems take no action. It is only later when these problems persist that they either visit a health facility. This was reported in thirteen of all FGDs. Reasons for this poor health seeking behaviour reported included cost, privacy issues and the long queues due to very few health workers serving large populations. Some adolescents in male FGDs reported “nosy” health workers who they thought asked many questions making them uncomfortable.

*You cannot come and tell a health worker to give you what you want, she will ask you a lot of questions and afterwards she will not even give you what you want. You may just find yourself failing to tell her what you want because she is asking you a lot of questions.* (Males in school)

Our results show that there were ‘under-the-table’ payments in health service delivery. In some cases unofficial payments were reportedly solicited which further made the services for adolescents hard to access because majority do not have money but depend on parents/guardians.

*When I get a problem, I go to the health facility. You can go there early and you sit in a queue for long then someone comes with their money e.g. 3000/=, which they give to the health worker and they tell them to go and get them medication. So they are worked on before you. Also if you go for things like drawing blood for tests, the health worker first asks for money to buy gloves and sometimes these could be available yet they don’t want to use them. So the one who has given them money is the one who is given drugs and then you: you could be given only Panadol: like 3 tablets or so and then you leave.* (Females out of school)

Worth noting is that some of the adolescents (when problems persisted), sought help from traditional healers or used herbs. This was reported in seven of the ten female FGDs and in two of the 10 male FGDs. In half of the FGDs, some adolescents reported that they sought advice from parents and peers when faced with such problems.

*One would rather go to a traditional herbalist than a health worker …because he will think that the health worker will tell others about his sickness but a traditional healer who is far where he is not known is where he goes*.(Males in school)

*You tell the person who is available: if you have your mum, you tell her. If you have your aunty, you tell her. Those who have their fathers, it is difficult for them to disclose to them because many fear telling their fathers feminine problems, they prefer mothers.* (Females in school)

In a few FGDs (6/20), adolescents also noted that they seek advice from more older people in the community while others who can afford go and buy medicines from drug shops or pharmacies (4/20 FGDs). Private health facilities (including clinics, pharmacies and drug shops) were the most accessible to the adolescents than public health facilities. Access in this case was beyond just the physical dimension.

*When we get problems, sometimes we tell our friends. Me I have a friend who has Candida, but she told us her friends and we inquired from an older person about it, who told us about some local medicine so that is what she is using. She had been to Kasangati health centre and they told her that the drugs are not there, they just wrote a prescription and they told her to buy the drugs from a clinic yet she had no money and no parents. So sometimes you can tell friend or neighbours, they may be of help.* (Females in school)

In this study, we also asked adolescents whether they went to the health facilities to seek for SRH information. In half of the FGDs, they reported to have never gone to the health facilities to seek SRH information. The reasons given were lack of information about the availability of these services at the health facilities while others feared to seek services in the same facilities with the older community members and with different sex of health workers.

*Another problem is that most of the health workers here are females (they laugh). Such ladies are easy to their fellow ladies but to us boys it is not easy. *(Males in school)

*The problem we have found as young men, you may get into a situation. You know young people of our age; they date as girlfriend and boyfriend. So he may have no condoms and he may not go to the health facility to pick them yet they are there; he is just shy. So he may have intercourse and he gets HIV/AIDS because he has acted shy saying “how will I be seen picking them?”* (Males in school)

Only in one quarter of the FGDs (5/20), adolescents reported ever seeking services at the health facilities for SRH. Among those who said yes, we investigated what their attitudes were about the services provided. In nearly all FGDs (19/20), the adolescents reported that they did not think they were a priority at the health facilities, hence found no need to go back for SRH information and services. In most FGDs (18/20) also adolescents reported that health workers were not friendly to them (at worst labelling them as rude) and barked at them. They further reported a lack of privacy at these facilities for them in seventeen of all FGDs while in nineteen of the twenty FGDs adolescents noted that where the services were not free, the cost was not affordable to them (19/20). The overall quality of SRH services at the facilities was reportedly of poor quality to most of them as reported in fifteen of twenty FGDs.

*Sometimes it is difficult at the health facility. You could go with your friends and you do not want them to know what is disease [STI] is bothering you. Then as you are explaining to the health worker calmly, some of them bark at you saying “speak louder.” But you are scared of speaking loudly because your friendly will hear, you continue narrating to him in a low tone and then he screams “which disease?… syphilis?” So this leaves you too ashamed in the consultation room. The health workers are too tough; they should reduce on their toughness.* (Males out of school)

*You could go there and you find that you are the only one of our age in the group and you are scared of saying what has taken you there.* (Males out of school)

Other reported attitudes were: non flexible opening and closing hours of facilities (5/20), lack of necessary drugs for STIs and other SRH problems (12/20), few health workers yet adolescents feared to open up to health workers of the opposite sex, and mistrusting of the health workers by the adolescents.

*If you are from school and you have gone to the health facility and you are putting on a uniform, they may work on you vary first because they know you are a student but if you go in casual wear like on weekends, they treat you like the rest of the people. And after telling the health worker your problem, they may tell another health worker that “this one has this kind of illness, this one has this kind of illness.” You came to seek treatment from the health workers but they just discuss about you and that becomes a problem.* (Females in school)

Only in six FGDs, four of which were female, were the adolescents happy with the quality of services at facilities.

### Preferred services for adolescents and modalities of their provision

Adolescents were asked which SRH services they wanted to be provided in their communities and facilities. Most of these preferred services reflect the adolescent health needs reported in the first part of the results. In half of the FGDs, adolescents reported a need for a dedicated teenage health centre equipped with youth friendly health workers and stocked medicines. Adolescents also wanted provision of adolescent counselling services and health education. In health facilities outside of the teenage centre, adolescents want separate services to ensure privacy for them.

*Now what I am saying is that we as the youth we should get a special day say like the weekend and we have counselling and guidance, it will help us.* (Males in school)

*We [the adolescents] need health talks because there are some problems we face when we totally have no idea on how to go about them. So we should be health educated such that we know what to do.* (Females out of school)

Regarding modalities of service provision, the adolescents preferred that services be available all the time (opening and closing hours), by younger health workers and of the same sex and in places that ensure privacy. The out-of- the school male adolescent FGDs preferred services to in an outreach form in the communities, at no cost and preferably with health workers not from the same area. Those in school preferred friendly health care workers and reduction in waiting time at the health facilities.

*Me I think there should be a private room for picking condoms (they laugh), so that when you go there you just get enter: the person there knows what you want and they don’t ask for money. But if you go when all the health workers work from there, when one asks you, you will be afraid of telling them what you want but if you just enter a room, the person you find there automatically knows what you have gone to pick from there.* (Males out of school)

*For me am 19 years but the years I have lived here, I have never seen people coming to offer youth counselling here. Yes as youths we normally get problems and we fear to visit the health facilities that health workers will harass us. We cannot tell our parents neither our friends because they will spread the information to the public, so we need youth counselling here in the community.* (Males out of school)

## Discussion

Our results clearly show that adolescents have SRH issues that need to be addressed. Adolescents in and out of school had SRH issues such as unwanted pregnancies, STIs, defilement, rape and substance abuse. Unique to the females was the issue of sexual advances by older and adolescents men. We also established that with regard to health seeking behaviour, most adolescents do not take any action at first until disease severity increase.

Adolescence is a unique age [34] whether in school or out-of-school. It is the environment and level of exposure that differed in this study. Our study highlights several challenges faced by adolescents. It is important to say that female adolescents are at greater risk and therefore more vulnerable than their male counterparts. Female adolescents face problems of rape, sexual abuse, unwanted pregnancies and defilement among others. Specifically, the issue of sexual advances by older men exposes them to several vulnerabilities. Our findings are in line with what literature advances i.e. vulnerability to STI and HIV infection [35,36], early marriage, dropping out of school [37]. On the other hand, a meta-analysis that used a nationally representative multicounty data identified that adolescent males are the most vulnerable group for STI including HIV [38]. This implies that when setting up adolescent specific interventions, gender sensitivity ought to be paid attention to. Also important to note that substance use and abuse was reported to be on the increase as is the case indeed with the wider society in the country and as such needed to be addressed hence forth. Agents of addictive dangerous substances such as tobacco, marijuana, target adolescents to boost their sales. It is important also to note that this is not a Ugandan problem alone but this trend has been observed elsewhere.

We further highlight SRH needs which would be solved by establishing adolescent friendly clinics with the standard of care and characteristics as recommended by adolescents [39]. With regard to health seeking behaviour, most adolescents do not take any action until illness severity increases. This has been evidenced in other studies in Burkina Faso, Ghana, Malawi and India where adolescents were highly unwilling to seek SRH services until the problem is severe such as STI services and HIV testing which are still under-utilized by these adolescents [40,41].

Further still, results resoundingly revealed that there was strong need for establishment of adolescent friendly centres that would provide the much needed SRH services. Adolescents expressed a great need for post abortion care services for females, and access to contraceptives as well as contraceptives education. Both male and female FGDs expressed a need to have youthful counsellors who they thought would best listen to and understand their problems. Issues highlighted often leave no choice but to have adolescents seek services from herbalists who often are more accessible socially and economically. Currently in Uganda, the issue of abortion is still sensitive but high on the agenda in public domain and civil society. Even though the legal framework appears not to be so restrictive on paper [42] there is a need to explore sex education options that are more acceptable to most stakeholders including those from different religious affiliations in Uganda.

The current health care service provision set up was reported to be less attractive to adolescents. The services largely lack privacy and have few trained health workers that can provide effective adolescent friendly services. This has also been evidenced in other studies [43,44]. Some initiatives have been tried in Uganda and appear successful such as the Naguru teenage centre. Expansion of such initiatives would contribute significantly to improving SRH of adolescents in Uganda and would be desired although the cost of such specialised centres may be a hindrance for a low income Country like Uganda. In line with our findings, the State of World Population report 2013, published by UNFPA highlights the main challenges of adolescents as unwanted pregnancies and its serious impacts on girls’ education, health and long-term employment opportunities. The report applies a multilevel ecological framework, which shows that adolescent pregnancies do not occur in a vacuum. They are the consequence of a combination of factors, including poverty, communities’ and families’ acceptance of child marriage, and inadequate efforts to keep girls in school [45].

One of the strength of this study was the categorisation of the different groups that helped us to make compulsions across these multiple categories. However, the limitation of this study was the definition of what constitutes SRH services, it is different in different contexts hence it makes it difficult to generalise the findings. This study was also limited in scope and coverage. Our study was conducted in Wakiso district, one of the 112 districts of Uganda at the time. While this was a formative study and a bigger study with national coverage would be good to undertake in future.

Conclusively, there is a need to focus on adolescent needs and services for both in and out-of-school adolescents with adolescent friendly services. Our study gives evidence of challenges and the need to address them. Opportunities for short and long term trainings for health workers should be explored. Training in post abortion care and providing adolescent friendly services need to be emphasized. Adolescent sensitive materials and literature need to be produced. Health facilities need to designate a corner or space where adolescent health is exclusively provided. Besides, stakeholders such as parents, teachers, and legislators need to pay attention to the rising substance use and abuse. Revitalising the Country’s formal and informal structures would go a long way in responding to this problem.
